# A twenty-eight-year laboratory-based retrospective trend analysis of malaria in Dakar, Senegal

**DOI:** 10.1371/journal.pone.0231587

**Published:** 2020-05-15

**Authors:** Mamadou Alpha Diallo, Aida Sadikh Badiane, Khadim Diongue, Linda Sakandé, Mouhamadou Ndiaye, Mame Cheikh Seck, Daouda Ndiaye

**Affiliations:** Department of Parasitology, Cheikh Anta Diop University, Dakar, Senegal; Johns Hopkins University Bloomberg School of Public Health, UNITED STATES

## Abstract

**Introduction:**

Health facility-based records offer a rich source of information to understand trends and changes in malaria cases over time. This study is aimed at determining the changes in malaria occurrence over the last 28 years, from 1989 to 2016 in Dakar, Senegal.

**Methods:**

Laboratory suspected and confirmed malaria records from 1989 to 2016 were reviewed from the laboratory registers of the Laboratory of Parasitology and Mycology of Aristide Le Dantec Hospital. Interrupted time series (ITS) analysis was used to estimate the changes by comparing malaria cases post-intervention (2006–2016) with that of the pre-intervention (1989–2005) period.

**Results:**

A total of 5,876 laboratory confirmed malaria cases were reported out of 29,852 tested cases, with total slide positivity rate (SPR) of 19.7%. Malaria case counts exhibited a fluctuating trend with major peaks occurring in the years 1995 and 2003 with SPR of 42.3% and 42.5%, respectively. Overall, a remarkable decline in the total number of laboratory confirmed malaria cases was observed over the last 28 years. *P*. *falciparum* was almost the only reported species, accounting for 99.98% of cases. The highest SPR was observed in the age group of under five years during the pre-intervention period while this shifted to the age group of 6–15 years old for the subsequent years. Two major malaria peak seasons were observed: one in September during the pre-intervention period and the other in November for the post-intervention period. The ITS analysis showed a dramatic decline of 83.6% in SPR following the scale-up of interventions in 2006.

**Conclusion:**

A remarkable decline in laboratory confirmed malaria cases in Dakar over 28 years was observed. The period of rapid decline in malaria SPR coincided with the scale-up in interventions beginning in 2006 with the introduction of ACTs, followed by the widespread introduction in 2008 of bed nets treated with insecticides. Robust surveillance data should be maintained in the context of malaria elimination efforts.

## Introduction

Malaria is widely distributed in tropical countries and still remains a major public health problem, particularly in sub-Saharan Africa. In 2017, over 219 million clinical cases and an estimated 435,000 deaths from *Plasmodium falciparum* infection occurred, primarily in young children from sub-Saharan Africa. With the exception of the WHO South-East Asia region, where the incidence of malaria continues to decrease (17 cases per 1,000 inhabitants exposed to the risk of malaria in 2010 against 7 per 1,000 in 2017, a decrease of 59%), all WHO regions recorded very modest progress, or even an increase in malaria incidence [1].

In Senegal, there is evidence of an overall decline in malaria prevalence among the general population over the last few decades [2]. Although many factors have acted in synergy to decrease the malaria endemicity in Senegal, the main reasons for this decline seem to be related to intervention efforts that have been in place since 2005 [3], as suggested by the faster rate of decline in malaria incidence since 2006. Senegal has built an effective national malaria control program (NMCP) based on strengthened management and well-defined plans. This strategic planning was critical in attracting partners and external financial resources [4] to facilitate the implementation of these interventions. The NMCP have implemented integrated intervention strategies including artemisinin-based combination therapy (ACT) in 2006, systematic diagnosis using rapid diagnostic tests (RDTs) in 2007, universal coverage with long-lasting insecticide-impregnated bed nets since 2008 (LLINs) [5].

Currently, malaria transmission, in Senegal, is characterized by very low transmission in the north, low transmission in the center, and high transmission in the south [6]. The success in malaria control in Senegal is illustrated by the substantial change in malaria epidemiology in the villages of Dielmo and Ndiop, located in the district of Sokone, in Fatick region, about 250 Km southeast of Dakar. In this longitudinal prospective study in a community living in rural areas with intense and perennial malaria transmission, malaria related indicators dramatically decreased from 1990 to 2012; the parasite rate dropped from 90% to 0.3% (meeting the WHO criteria for malaria pre-elimination) [7]. On the other hand, in Dakar, the capital city, the transmission is very heterogeneous, representing a separate transmission zone: the urban malaria [8]. Historically it was thought that there was no malaria transmission in the downtown area and that the infections occurred only in the suburbs or inland. However, it has been demonstrated that malaria transmission occurred in autochthonous people who had not been outside Dakar for at least one year [9].

Robust surveillance data is essential to evaluate the impact of existing intervention strategies, to inform future malaria elimination activities and for certification of elimination. Before the reorganization of the NMCP in 2005, insufficient malaria data was collected at the country level to facilitate analysis of malaria prevalence trends and the intervention mechanisms employed each year. In this context, health facility-based prevalence studies can provide useful indicators [10] and enhance understanding of the situation of malaria as previously reported elsewhere in Africa [11–13]. Since 1988, the laboratory of Parasitology and Mycology of Aristide Le Dantec hospital, malaria microscopy was performed and data recorded for all suspected patients, long before the integrated interventions were introduced by the NMCP.

The aim of the present study was to analyze the factors associated with the changes in malaria slide positivity rate (SPR) over 28 years (1989–2016) using laboratory record indicators and examine the relationship between this rapid impact and malaria interventions and the times at which they were implemented. Attempts were made to analyze the implications of climate factors–especially rainfall, urbanization process and other factors that could synergistically affect malaria prevalence over the last three decades. The results are discussed in line with global goals for malaria control and elimination.

## Methods

### Ethics

This study involved analysis of routinely available and individually anonymized data at health facility, and received approval from the Senegalese National Ethic Committee of Ministry of Health.

### Study area

Dakar is the capital city of Senegal and the population is estimated to be 3,700,000, representing 20% of the Senegalese population. It is located at 14°40'20" North, -17°25'22" West (the westernmost point of Africa), on the southeastern side of the Cape Verde Peninsula in the Atlantic Sudan zone. The altitude does not exceed 104 m. There are two seasons a year: a long dry season of almost eight months from November to May and a rainy season from June to October. The first rains generally occur at the end of June, and the last occur at the beginning of October [9].

Aristide Le Dantec University Hospital is a large hospital, and serves as one of the main treatment centers for the millions of people who reside in Dakar and patients coming from rural Senegal and neighboring countries. The Hospital offers general medical services in all major disciplines. It is also a teaching hospital and health research center for training students.

The Laboratory of Parasitology and Mycology (LPM) at Aristide Le Dantec University Hospital offers a wide range of investigations including diagnosis and identification of parasites and fungi in clinical samples. Besides performing routine diagnosis tasks, the laboratory is the national reference laboratory for the national malaria control program (NMCP) in malaria diagnostics. The laboratory has established a malaria slide bank to enable quality in-service and pre-service malaria microscopy trainings, competency assessment of microscopists, laboratory mentorship programs, and national and regional malaria microscopy External Quality Assessment (EQA) programs. Most of the laboratory personal have more than five years’ experience in malaria microscopy; Six of them are certified level 1 WHO malaria microscopists. Since 2016, the LPM hosts the WHO External Competence Assessment of Malaria Microscopy (ECAMM) course for Africa’s Francophone countries.

### Study design and data collection

This study was carried out based on primary data from laboratory registers at the LPM at Aristide Le Dantec Hospital for both inpatients and outpatients. Twenty-eight-years of malaria data was collected from laboratory record books from 1989 to 2016. The parameters recorded included date of examination, gender, age, relevant clinical information and microscopy result (parasite species). The cases with incomplete records of important variables were excluded.

Since 2007, the Senegal NMCP follows the WHO recommendations for parasite confirmation either by microscopy (at the hospital level) or malaria RDT (at health post facilities) in all patients with suspected malaria before treatment is administered.

Before 2006, in the absence of biological diagnosis, all fever cases were considered as malaria and treated with chloroquine. There was no harmonized procedure for malaria diagnosis at the country level. At Aristide Le Dantec Hospital, however, the microscopic examinations of thick and thin blood smears were routinely offered since 1988 in febrile patients with clinical signs suggestive of malaria. Therefore, in this study, malaria case was defined as microscopically confirmed cases. We assumed that all tested cases were first suspected beforehand.

The laboratory followed the standard operating procedures (SOPs) for Giemsa preparation and staining. Briefly, thick and thin smear were made from capillary blood sample collection, and stained using 10% diluted Giemsa, and blood film examination for malaria parasite detection by at least two senior malaria microscopists. In the study, at least 100 high-power fields were observed before a slide was declared negative. Rainfall and temperature information for this time-period were extracted from the ANACIM (National Agency for Civil Aviation and Meteorology) website and to capture the relationship with the annual SPR. Rainy years were defined as years when the annual rainfall exceeded 500mm.

### Statistical methods

#### Group comparisons

A Chi-square test was used for comparing differences in categorical data. Confirmed malaria cases were compared between age groups, and between outpatient and inpatients before and after the intervention and tested statistically for evidence of changes.

#### Descriptive analysis of trends

A simple descriptive interpretation of trends in SPR were applied by visually inspecting the plotted trend.

#### Interrupted time series (ITS)

This approach was used to analyze the longitudinal laboratory recorded data and evaluate the impact of introducing the scale-up of interventions on SPR, taking into account background secular trends and climatic factors (rainfall). The specification of the linear regression model to be analyzed for each outcome is:

$$Y_{t}=\beta_{0}+\beta_{1}\;time_{t}+\beta_{2}\;level_{j}+\;\beta_{3}\;trend_{jt}+\;\varepsilon_{jt}$$ (1)

Where *Yt* denotes the SPR (outcome) in year *t*; *time* is a continuous variable indicating the time in years at time *t* over the entire period; *level* is a dichotomous indicator variable for the scale-up intervention, equal to zero before the start of the intervention and equal to one after the start of the intervention; *trend* is a continuous variable counting the number of years after the scale-up intervention starts, and equal to zero before the intervention starts. Finally, *εt* is an error term at time *t* that represents random variability not explained by the model. *β0* represents the existing level at time 0 (baseline level or intercept); *β1* estimates the existing trend in the SPR per year over the entire period; *β2* estimates the level change in the SPR per year immediately after the scale-up intervention; *β3* is the coefficient that captures the change in trend in the SPR after the start of the intervention. The sum of *β1* and *β3* is the intervention-period slope. By controlling for baseline level and trend, the model estimates level and trend changes associated with the start of the intervention. The coefficients are illustrated graphically in Fig 2 and describe the analysis undertaken using Eq 1. The Durbin-Watson statistic along with autocorrelation function (ACF) and partial autocorrelation function (PACF) were used to test for the presence of autocorrelation. When this was detected, the final model was adjusted accordingly. Data were entered and cleaned using Microsoft Excel Version 16.33, and analyzed using RStudio version 1.2.5001 and the ‘patrick-eng/ITS.analysis’ package.

## Results

### Description of data and overall malaria occurrence

**Table 1**describes the general characteristic of the study population. Over a period of twenty-eight years, 29,852 patients were admitted and tested for malaria in the laboratory. Out of these, 18,923 (63.3%) were admitted as inpatients. This distribution was similar between pre-intervention and post-intervention periods (60.0% and 69.3% were respectively inpatients). The admission rate (number of admitted patients per year) were similar between the two time periods (1123 per year and 1108 per year respectively). Higher malaria admission rates (n > 1000 per year) were recorded between years 1996 and 2010. This proportion decreased progressively in the subsequent years (**Fig 1**).

**Fig 1:**
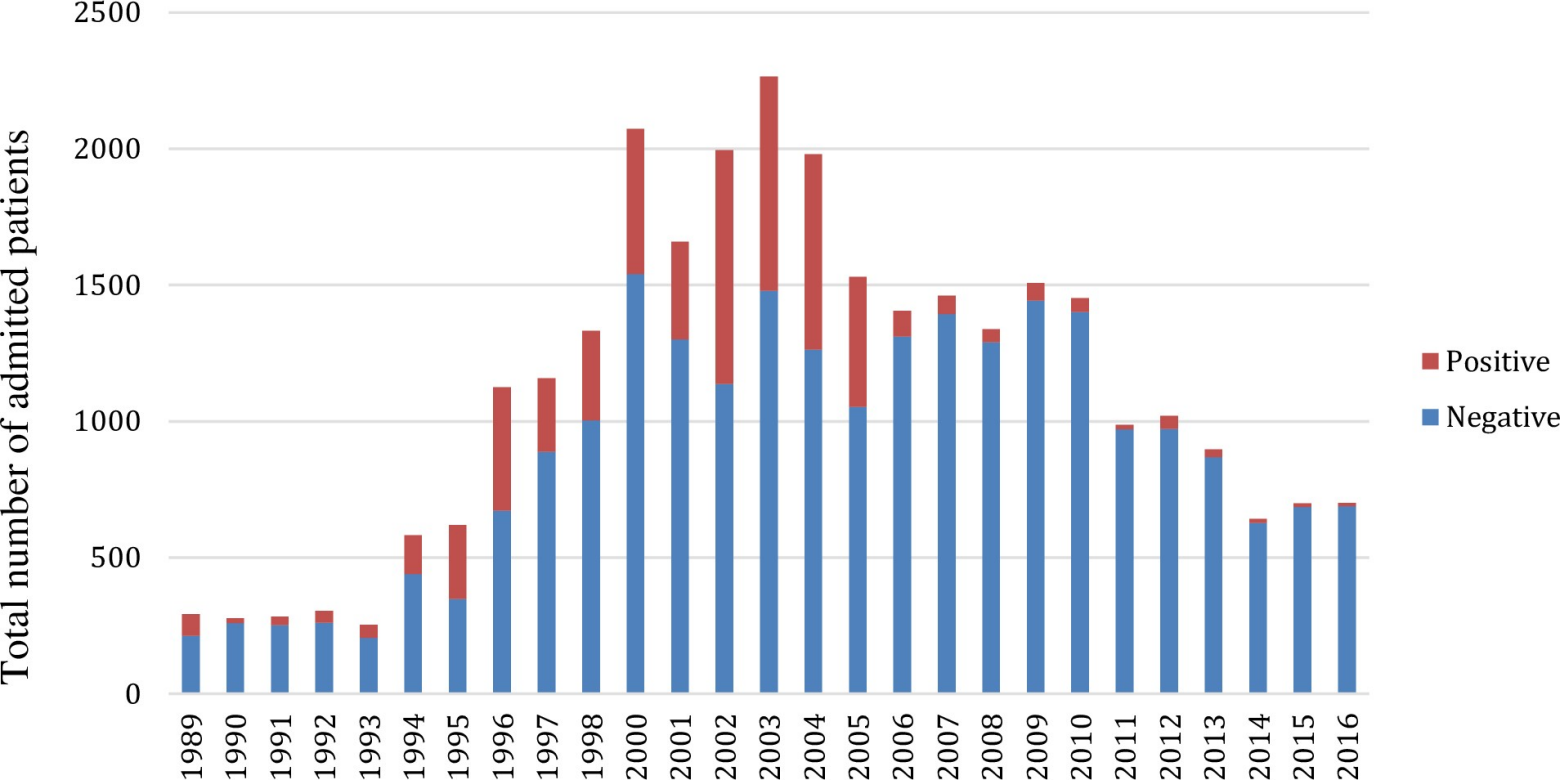
Annual trend of tested patients highlighting number of confirmed cases along with number of negative cases from 1989 to 2016.

**Table 1:** General characteristics of the study population during the pre-intervention and post-intervention periods.

	Pre-intervention (1989–2005)	Post-intervention (2005–2016)
**Total admission for malaria diagnosis *n***	17960	12191
**Annual admission for malaria diagnosis *n***	1123	1108
**Median age *n***	30 y	26 y
**Admission across age groups *n* (percentage)**		
< 5 y	218 (1.2%)	1079 (8.9%)
5–15 y	374 (2.1%)	1206 (9.9%)
> 15 y	17368 (96.7%)	9906 (81.3%)
**Total slides positive *n* (annual SPR)**		
all ages	5415 (30.0%)	1461, (3.8%)
< 5 y	79 (36.2%)	29, (2.7%)
5–15 y	123 (32.9%)	58, (4.8%)
>15 y	5213 (30.0%)	375, (3.8%)
***x***^***2***^	5.318689	7.033004
**p-value**	0.0699941	0.0297032
**Admission across outpatients and inpatients**		
outpatients (%)	7020 (40.0%)	3719 (30.7%)
inpatients (%)	10538 (60.0%)	8385 (69.3%)
positive in outpatients (SPR)	2569 (36.7%)	185 (5.0%)
positive in inpatients (SPR)	2719 (25.8%)	276 (3.3%)
***x***^***2***^	233.219846	19.915447
**p-value**	0.0000000	0.0000474
**sex ratio (male/female)**	7820/9785	6182/5923
positive in males (SPR)	2382 (30.8%)	247 (4.0%)
positive females (SPR)	2929 (30.3%)	212 (3.6%)
***x***^***2***^	0.57	1.44
**p-value**	0.449	0.231

**Fig 2:**
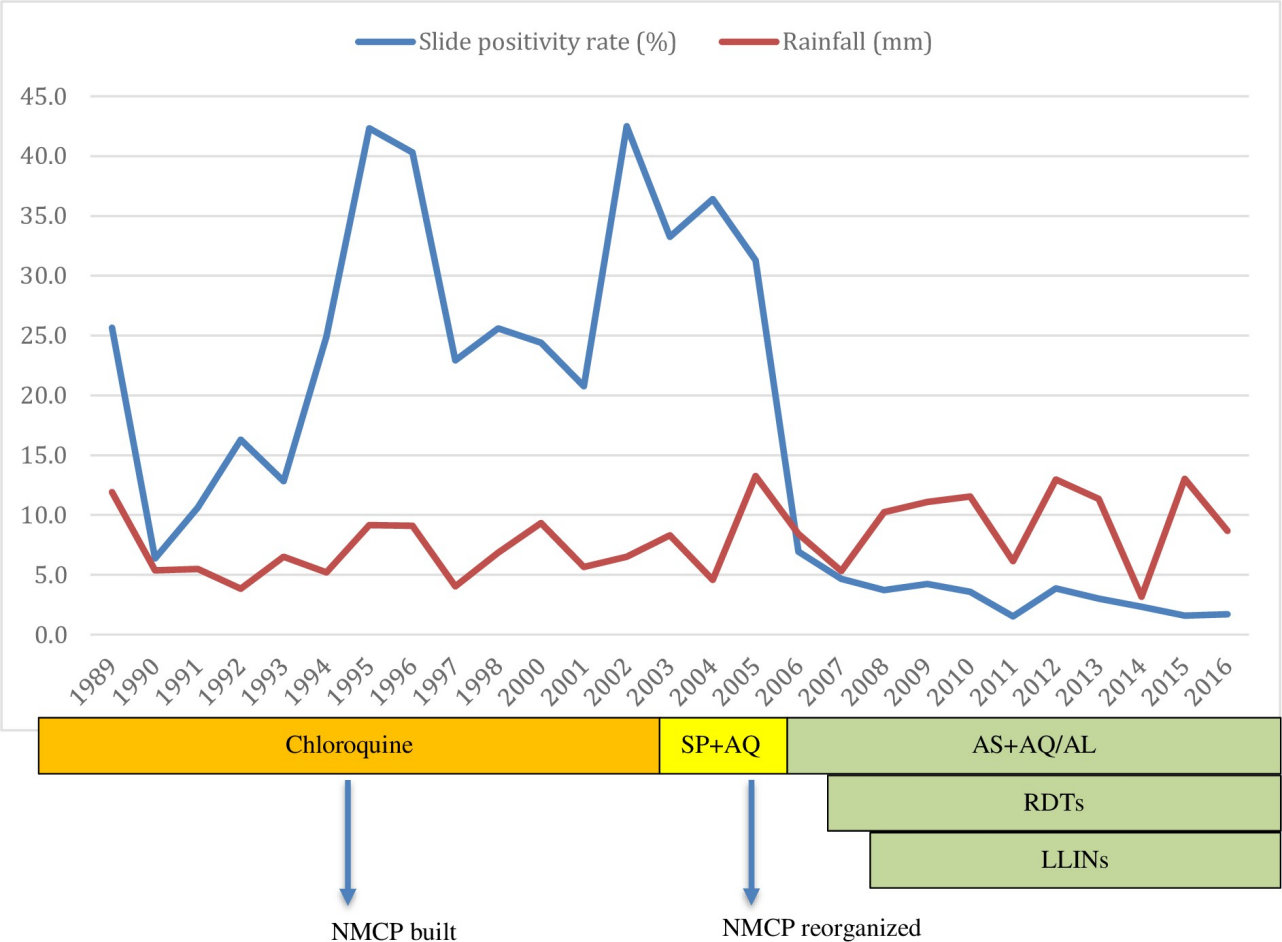
Yearly proportion of malaria slide positivity rate (SPR) and interventions deployed from 1989 to 2016; rainfall is plotted with a scale of 1/50 over the entire period.

Out of the 29,852 tested patients, 5,876 were confirmed malaria cases by microscopy–slide positivity rate (SPR) of 19.7%. *Plasmodium falciparum* accounted for 99.98% of malaria cases. There was only one *P*. *vivax* case, from a Mauritanian patient. The highest proportions of confirmed cases were recorded between 1994 and 2005 where the annual SPR exceeded 20.0%. There were two major peak years: 1995 and 2002 with SPR of 42.3% and 42.5% respectively. The lowest level was recorded in 2011 with a SPR of 1.5% (**Fig 2**). The SPR was significantly higher in outpatients than in inpatients in both the pre-intervention period (36.7% vs 25.8% respectively, p<00.1) and the post-intervention period (5.0% vs 3.3% respectively, p<00.1) as shown in **Table 1**. Of the total number of patients examined, the sex ratio (male/female) was 0.89. The SPR was not significantly different in the two groups for the two time periods (p = 0.449 and p = 231 respectively).

### Trends in malaria occurrence

Malaria showed a fluctuating trend during the twenty-eight years of the study period, from 1989 to 2016. The number of annually tested and confirmed malaria cases generally increased between 1989 and 2003 while numbers decreased between the years 2003 and 2016. The number of confirmed cases showed a sharp rise for five consecutive years, from 1990 to 1994. From 1994 to 2001, the number of cases went into decline, before rising to a second peak in 2002. After a slight decrease between 2002 and 2005, a steady decline followed in 2006 (a decrease by 78% in one year), reaching < 5% from 2007 onwards. Since 2007, there has been an overall trend towards declining malaria cases, with a small rebound in 2012 when the SPR was 3.9% (**Fig 2**). Overall, a trend towards an increasing proportion of non-malaria cases and a decreasing number of laboratory confirmed cases of malaria (**Fig 1**).

### Time series analysis results

**Fig 3:**
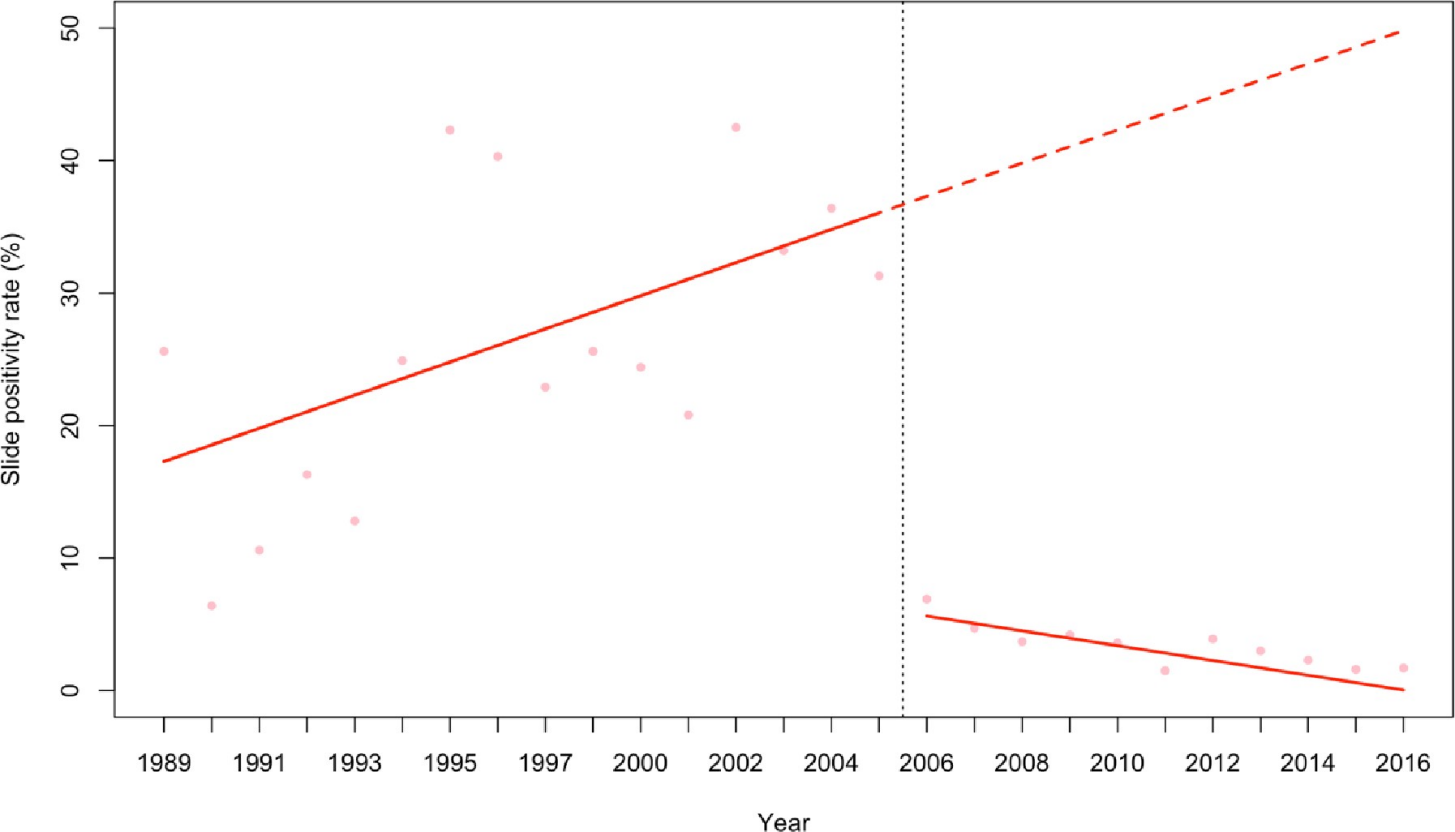
ITS model showing the trends in slide positivity rate during the pre-intervention and post-intervention periods. Fitted lines illustrate the linear trends from model predictions of malaria SPR controlling for rainfall and controlling for autoregressive and moving average effects (solid red line) and observed malaria SPR (pink dots). The hypothetical trend in the absence of intervention is also shown (dashed red line).

The predictions from the best fitting model that adjusted for rainfall while controlling for autoregressive and moving average effect within the data are shown in **Fig 3**. We included linear trend lines fitted to estimate the average yearly changes in malaria SPR. Before the scale-up of interventions, the model showed a significant upwards trend (p<00.1). After the scale-up of interventions, there was a sustained decrease in the average annual SPR; SPR was 29% lower (p<0.001) compared to the pre-intervention period, which represents an 83.6% decrease in the SPR between the two time periods. Since then, a significant decreasing trend was observed (p = 0.002) with an annual reduction in SPR of 1.8%. Rainfall did not significantly affect the SPR outcome in the model predictions (p = 0.1744). When rainy years were incorporated in the segmented regression model as an indicator variable, it did not significantly affect the SPR outcome in that particular years (p = 0.177).

### Age distribution of cases and change over time

Individuals over 15 years of age accounted for more than 80% of all admitted patients during both pre-intervention and post-intervention periods (**Table 1**). The SPR in different age groups was significantly different during the post-intervention period only (pre-intervention p = 0.07; post-intervention p = 0.03). Notably, the SPR was slightly higher in children under five years old (36.7%) for the pre-intervention period (p = 0.07) while after intervention the highest SPR was observed among 6–15 years old (4.9%).

### Seasonal variations in malaria positivity rate

**Fig 4:**
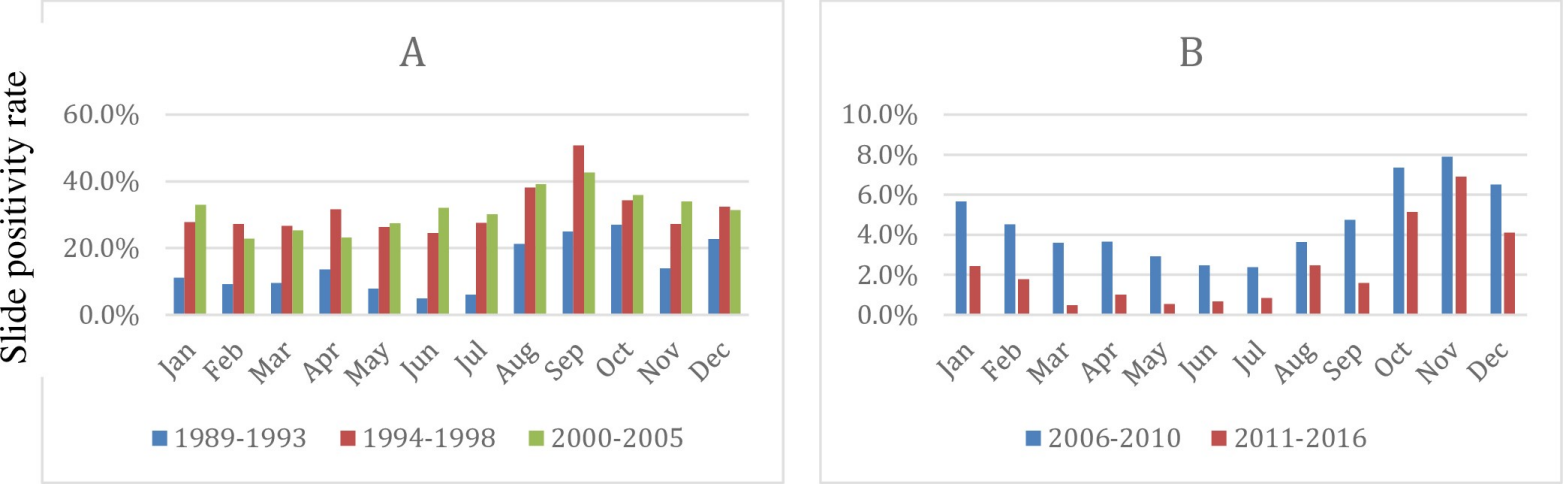
Temporally aggregated monthly SPR showing shifted peaks of malaria transmission from A) September during the pre-intervention period (1989–2005) to B) November in the post-intervention period (2006–2016).

Malaria SPR showed a strong seasonality. However, there was a remarkable difference in the peak seasons between the two time periods, 1989–2005 and 2006–2016 respectively. Before 2006, the most cases were observed from July to November with a peak in September; in the later time period cases peaked in November and covered September to January (**Fig 4**).

## Discussion

Monitoring malaria can be challenging in Africa due to the lack of basic population and health data [14,15]. Here we reported the relevance of analyzing routinely collected malaria data from laboratory registers to understand the malaria situation with regard to its trend.

In our data, the number of tested cases as well as positive cases increased gradually between 1990 and 2000 and it decreased during the subsequent years. Similar patterns of increase and decrease in overall malaria burden over the same period have been observed by other studies [16–18], suggesting that these changes were taking place across the entire country. However, some differences between areas were also observed. In our series, the most rapid decline was observed between 2005 and 2006 when the SPR decreased from 31% to 7%, coinciding with the scale-up of interventions. In contrast, in Dielmo, this decrease was gradual with an annual prevalence under 5% being achieved 2 years later than in Dakar– 2009 compared to 2007. At the same time, the 2010–2011 Demographic Health Survey (DHS) showed a regression of almost 50% of parasite prevalence between 2008 and 2010 among children under 5 years old in accordance with the NMCP data reports [3]. From 2007 onwards, malaria cases were higher in age group 5–25 years old in contrast with the pre-intervention period where the youngers were the most concerned; this might be due to the fact that many interventions targeted the under five years old group and this could make the older group more susceptible [7].

The success of malaria control in Dakar, Dielmo and elsewhere in Senegal is largely a result of the policies that have been implemented over the years in the country. In 1995, the NMCP was created [19] and has actively participated in the development of the first national strategic plan for malaria control (1995–2000). Unfortunately, chloroquine-resistant *P*. *falciparum* spread quickly; by mid-1995, almost half of *P*. *falciparum* clinical infections harbored the chloroquine-resistance associated *Pfcrt* mutation [7]; these treatment failures were the probable cause of the increased incidence in malaria morbidity and mortality [19,20]. Despite its inefficacy, this molecule was still used until 2003 when the resistance reached its highest level [20]. As such, the NMCP reviewed treatment policy, replacing chloroquine by sulfadoxine-pyrimethamine plus amodiaquine (SP+AQ) from November 2003 to May 2006. Following WHO recommendations, ACTs were adopted and artesunate plus amodiaquine was introduced from June 2006 onwards [7]. The implementation of long-lasting insecticidal-treated bed nets (LLINs) has contributed to halving the mortality rate due to malaria since 2000 in Senegal and elsewhere in sub-Saharan Africa [21].

The NMCP results were made possible thanks to, largely, the technical and scientific support from academic and research institutions. The Cheikh Anta Diop University of Dakar (UCAD) noted for the first time the existence of resistance to chloroquine. This led to a policy review process in 2003 and the adoption in 2006 of the artesunate-amodiaquine combination as first-line treatment for uncomplicated malaria cases. Thus, the key NMCP interventions were based on data from operational research [22].

Beyond the successive actions that have been taken over the years, one should consider the importance of the change in environmental factors in the incidence of malaria during the same time frame. For instance, rainfall is known to be a major factor for the development of mosquito vectors [23]. Rainfall decreased by 50% in Dakar between 1950 and 2000. Since the early 2000s, however, rainfall appears to have increased significantly [24]. The annual rainfall data for the period 2005–2010 showed a potential for a sustained favorable environmental for malaria transmission, notwithstanding the decreasing trend of malaria reported by our study as well as NMCP reports [6]. Similarly, in The Gambia, changes in rainfall caused some fluctuations in malaria from year to year, but did not account for the progressive reduction that was recorded [25]. In our ITS model, the rebound peak observed in 2012 could not be explained by the heavy rains that caused important flooding in Dakar in this year. At the same time, resurgences of malaria transmission were observed in the Dielmo village [21] and in Thiès [26], the latter being an urban area, sharing a similar epidemiological pattern as Dakar. However, it is likely that this resurgence in malaria would have been even more marked in the absence of the interventions which were maintained.

Additional factors that have not been measured, or are difficult to quantify, may be important in this regard. This may include improved economic status and the use of hospital facilities [27]. In addition, there are benefits of urbanization in Dakar, since the proportion of the low risk population increased while urbanization progressed [8]. For instance, the overall observed malaria prevalence in Dakar in 2008 was 1.78% and a Geostatistical ZIB model showed that living in urban areas reduces the parasitemia odds by 81%. [28]. A cross-sectional study conducted in 2013 showed areas in Dakar where the prevalence rate in children is above 5% [29] while the average for agglomeration is close to 2% [30]. Sanitation could have an impact in that it prevents the production of mosquito breeding sites. This was the case in Deggo, a district of Pikine which used to be a malaria hotspot each year after the rainy season. The building of roads, canals and dredging allowed the disposal of wastewater and rainwater. This has led the district to move from the red zone (incidence > 15/1000 inhabitants) in 2012 to an intermediate level of transmission after the sewage works [2]. With all these factors defined above, it appears clearly that robust surveillance data is important to inform malaria elimination activities and for certification.

### Limitations

Study limitations include the use of slide positivity rate (SPR) among hospital admitted cases from retrospective data as an index of transmission intensity which may be biased by many factors. The index of suspicion in the denominator may be very variable since there was harmonized procedure in the clinical suspicion of malaria. On the other hand, there was certainly considerable number of patients who were not tested and who were treated with antimalarials based on clinical diagnosis alone. Similarly, considerable number of cases did not present to the to the health facility. In addition, there were some incomplete registrations and in this record review. Specifically, the year 1999 was not included as no data was available for this year.

Limitations of ITS analysis include difficulty in analyzing the independent impact of separate components of a program that are implemented close together in time; this was the case in our series where SP+AQ closely preceded the main intervention in 2006; and RDT use followed one year later. In addition, it is challenging to model many factors which are difficult to measure or not available (improved sanitation, urbanization process, economic status changes, operational research impact, etc.). Lastly, our data were taken from a single setting and therefore, our findings may not be generalizable across the country.

## Conclusions

Laboratory-based data can be a good reflection of the national malaria endemic situation. In this study we found that the malaria infection rate has generally declined in Dakar, from 1989 to 2016. Malaria reduction seemed to be associated with the scale-up of interventions since 2006 with the introduction of ACT, RDTs, LLIN and IRS.

However, despite substantial reductions in malaria transmission in Dakar, residual malaria transmission continued and older children were increasingly vulnerable to the disease, highlighting the need for expanding control interventions to all age groups. Maintaining control measures will be essential but will require novel strategies, including robust surveillance data to track the rapidly evolving landscape of malaria transmission.
